# Anosognosia in dementia with Lewy bodies: a systematic review

**DOI:** 10.1590/0004-282X-ANP-2020-0247

**Published:** 2021-05-08

**Authors:** Victor CALIL, Felipe Kenji SUDO, Gustavo SANTIAGO-BRAVO, Marco Antonio LIMA, Paulo MATTOS

**Affiliations:** 1 Instituto D’Or de Pesquisa e Ensino, Rio de Janeiro RJ, Brazil. Instituto D’Or de Pesquisa e Ensino Rio de Janeiro RJ Brazil; 2 Universidade Federal do Rio de Janeiro, Faculdade de Medicina, Programa de Pós-Graduação em Clínica Médica, Rio de Janeiro RJ, Brazil. Universidade Federal do Rio de Janeiro Universidade Federal do Rio de Janeiro Faculdade de Medicina Programa de Pós-Graduação em Clínica Médica Rio de Janeiro RJ Brazil; 3 Universidade Federal do Rio de Janeiro, Hospital Universitário Clementino Fraga Filho, Departamento de Neurologia, Rio de Janeiro RJ, Brazil. Universidade Federal do Rio de Janeiro Universidade Federal do Rio de Janeiro Hospital Universitário Clementino Fraga Filho Departamento de Neurologia Rio de Janeiro RJ Brazil; 4 Fundação Oswaldo Cruz, Rio de Janeiro RJ, Brazil. Fundação Oswaldo Cruz Fundação Oswaldo Cruz Rio de Janeiro RJ Brazil; 5 Universidade Federal do Rio de Janeiro, Instituto de Ciências Biomédicas, Programa de Ciências Morfológicas, Rio de Janeiro RJ, Brazil. Universidade Federal do Rio de Janeiro Universidade Federal do Rio de Janeiro Instituto de Ciências Biomédicas Programa de Ciências Morfológicas Rio de Janeiro RJ Brazil; 6 Universidade Federal do Rio de Janeiro, Instituto de Psiquiatria, Rio de Janeiro RJ, Brazil. Universidade Federal do Rio de Janeiro Universidade Federal do Rio de Janeiro Instituto de Psiquiatria Rio de Janeiro RJ Brazil

**Keywords:** Lewy Body Disease, Dementia, Metacognition, Doença por Corpos de Lewy, Demência, Metacognição

## Abstract

**Background:**

Anosognosia, i.e. lack of awareness of one’s own symptoms, is a very common finding in patients with dementia and is related to neuropsychiatric symptoms and worse prognosis. Although dementia with Lewy bodies (DLB) is the second most common form of degenerative dementia, literature on anosognosia in this disease is scarce.

**Objectives:**

This paper aimed to review the current evidence on anosognosia in patients with DLB, including its prevalence in comparison with other neurological conditions, its severity and anatomical correlations.

**Methods:**

Database searches were performed in PubMed, Web of Knowledge and PsycINFO for articles assessing anosognosia in DLB. A total of 243 studies were retrieved, but only six were included in the review.

**Results:**

Potential risk of selection, comparison or outcome biases were detected in relation to all the studies selected. Most of the studies used self-report memory questionnaires to assess cognitive complaints and compared their results to scores from informant-based instruments or to participants’ cognitive performance in neuropsychological tasks. Subjects with DLB had worse awareness regarding memory than healthy older controls, but the results concerning differences in anosognosia between DLB and Alzheimer’s disease (AD) patients were inconsistent across studies. Presence of AD pathology and neuroimaging biomarkers appeared to increase the prevalence of anosognosia in individuals with DLB.

**Conclusion:**

Anosognosia is a common manifestation of DLB, but it is not clear how its prevalence and severity compare with AD. Co-existence of AD pathology seems to play a role in memory deficit awareness in DLB.

## RESUMO

**Introdução:**

Anosognosia, i.e. a perda da consciência dos próprios sintomas, é um achado muito comum em pacientes com demência e está relacionada a sintomas neuropsiquiátricos e a pior prognóstico. Embora a doença por Corpos de Lewy (DCL) seja a segunda demência degenerativa mais comum, há pouca evidência sobre anosognosia nessa doença.

**Objetivos:**

Este artigo teve como objetivo revisar a evidência disponível sobre anosognosia em pacientes com DCL, incluindo sua prevalência em comparação a outras condições neurológicas, gravidade e correlações anatômicas.

**Métodos:**

Foram feitas buscas nos bancos de dados PubMed, Web of Knowledge e PsycINFO por artigos que avaliassem anosognosia na DCL. Um total de 243 estudos foi encontrado, mas apenas 6 foram incluídos nesta revisão.

**Resultados:**

Potenciais riscos de viés de seleção, comparação ou resultado foram encontrados em todos os estudos selecionados. A maior parte dos estudos utilizou questionários de memória preenchidos pelo próprio paciente e os comparou a resultados de instrumentos preenchidos por informantes ou à performance cognitiva em tarefas neuropsicológicas. Indivíduos com DCL têm pior consciencia de memória do que idosos saudáveis, mas os resultados tocantes à diferença de anosognosia entre DCL e doença de Alzheimer (DA) são inconsistentes entre estudos. A presença de achados patológicos e de neuroimagem de DA parece aumentar a prevalência de anosognosia entre pacientes com DCL.

**Conclusão:**

Anosognosia é uma manifestação comum da DCL, mas não é possível afirmar como sua prevalência e gravidade se comparam à DA. A coexistência de achados patológicos de DA parece influenciar a consciência de déficits de memória na DCL.

## INTRODUCTION

Babinski^1^ coined the term “anosognosia” (from Greek: *a-* = without; *nosos =* disease; *gnosis* = knowledge) to describe patients with hemiplegia who were unaware of their deficits. Since then, the concept has evolved to encompass all conditions in which recognition of one’s own symptoms or disorders is either reduced or eliminated^2^. It is a common finding in neurological practice, especially when dealing with patients with dementia, and it may impact quality of life and prognosis^3^. In fact, anosognosia for cognitive impairments has been correlated with increased prevalence of psychotic symptoms^4^, apathy^5^, poorer treatment compliance and increased risk of dangerous behaviors^6^. Also, among professional caregivers, the burden is greater when caring for individuals with this clinical feature^7^.

The most widely accepted theoretical model for anosognosia is the Cognitive Awareness Model (CAM)^2^. This framework implies that the experiences previously had by an individual are turned into semantic data, which are stored in a database. Cognitive awareness comes from comparison between this information and one’s performance in an ongoing task. This process is not unimodal, but occurs in specific modules for different domains (visual, motor, etc.), which helps to explain the existence of different modalities of awareness.

This idea of various types of awareness is reinforced by the concept of “objects of awareness”, defined as the mental or physical states in relation to which awareness is being assessed, as postulated by Marková et al.^8^. In this framework, one can be aware not only of measurable loss of function (e.g. hemiparesis or hemianopia), but also of subjective psychiatric symptoms, such as anxiety and hallucinations. Accordingly, whereas anosognosia in many neurological diseases affects one single aspect (e.g. in cortical blindness), subjects with dementia may be unaware of one or multiple cognitive domains, such as memory, executive functioning, language or psychiatric symptoms^2^.

Given the wide range of types of anosognosia, many methods have been developed to assess awareness in dementia, but none embraces all cognitive domains^9^^,^^10^. Since no harmonization of protocols has been proposed for research in this field, methodological heterogeneity across studies is substantial. This includes use of different conceptual strategies to measure the outcome (clinician’s judgment, discrepancies between reported and observed cognitive performances, and differences between patient’s and caregiver’s reports) and the choice of cognitive domains. In addition, a multitude of terms have been used to define the same phenomenon: besides the most appropriate label “anosognosia”, several other expressions like “unawareness”, “lack of insight” and “denial” are also frequently found in the literature^11^.

As expected from such heterogeneity, longitudinal studies about anosognosia in dementia have yielded inconsistent results regarding its course. This might also be attributable to variations in the duration of follow-ups^12^^,^^13^^,^^14^. There is, however, a tendency towards worsening of awareness with progression of the disease, with high interindividual variability^15^.

Among the many cognitive domains, self-awareness of memory has been the most extensively investigated. Its anatomical basis has been explored using different techniques of neuroimaging, including functional magnetic resonance imaging (MRI), MRI volumetry and FDG-PET. Most of the evidence points towards an important role for midline structures, particularly the prefrontal cortex (orbitofrontal and medial prefrontal cortex) and the anterior and posterior cingulate cortex^16^^,^^17^^,^^18^^,^^19^. However, more posterior regions, such as the medial temporal and parietal cortex and the hippocampus, have also been implicated^20^^,^^21^. Regarding laterality, many studies have suggested that there is greater involvement of structures on the right side^22^^,^^23^.

Despite evidence that levels of awareness may differ substantially among different etiologies for cognitive impairment, the data available have been heavily centered on subjects with Alzheimer’s disease (AD). Anosognosia is present in most patients with AD^12^^,^^14^^,^^24^, particularly regarding memory and appraisal of their own ability to perform activities of daily living^14^^,^^25^. It is noteworthy that this phenomenon has been reported to be even more severe in cases of behavioral variant frontotemporal dementia (bvFTD)^26^^,^^27^^,^^28^.

Although dementia with Lewy bodies (DLB) has been considered to be the second most common form of degenerative dementia^29^, few studies have evaluated anosognosia in this condition. Anosognosia may be particularly troublesome in DLB, as it may hinder treatment of the neuropsychiatric and motor symptoms that are common in this condition, thus worsening both the quality of life of patients and the burden of caregivers.

The aim of this paper was to review the current evidence regarding anosognosia in relation to different objects of awareness in patients with DLB, including its prevalence (compared with normal controls and AD patients), its severity and anatomical correlations.

## METHODS

### Literature search

On April 12, 2020, searches were performed on PUBMED, Web of Knowledge and PsycINFO using combinations of the following terms: (Diffuse Lewy Body Disease) OR (Lewy Body Dementia) OR (Lewy Body Disease) OR (Dementia with Lewy Bodies) OR (Cortical Lewy Body Disease) OR (Lewy Body Disease, Cortical) OR (Lewy Body Type Senile Dementia) OR (Lewy Body Disease, Diffuse) OR (Dementia, Lewy Body) AND (anosognosia) OR (awareness of deficit) OR (awareness of disease) OR (insight) OR (denial) OR (metamemory) OR (meta-memory) OR (metacognition).

The Medical Subject Headings database (MeSH) and the APA thesaurus were used to identify the index terms, In addition, entry terms and synonymous free text were included to enhance the sensitivity of the strategy. No limit was placed on the field or date of publication of the articles. The recommendations of the PRISMA statement were followed in this review. References of the studies selected were hand-searched for any eligible articles that had not been retrieved through the database searches. Authors with prominent work in the field were contacted by e-mail for possible relevant studies not published in indexed journals.

### Eligibility criteria

Studies were included if both of the following criteria were fulfilled: 1) the studies assessed older subjects (age ≥ 60 years old) with DLB diagnosed in accordance with specialist consensuses, i.e. DSM-5 or DLB Consortium; 2) subjects with DLB were compared with normal controls or patients with AD or other neurological conditions, regarding the presence and severity of anosognosia. There was no restriction on objects of awareness. Posters, case reports, reviews, comments and lectures were not included in this review.

### Data selection and extraction

Studies were independently screened for the eligibility criteria by two of the researchers (V.C. and G.S.B.). Any disagreements were resolved by reaching a consensus among the entire team of authors. Data on the sociodemographic aspects of the samples (mean age, schooling and gender), characteristics of the studies (design, setting and sample size), diagnostic criteria, prevalence of anosognosia and scores from instruments used to assess anosognosia were independently extracted by the same authors.

### Quality assessment

In parallel to the data extraction process, the risk of biases among the studies selected was assessed using the Newcastle-Ottawa scales for nonrandomized studies^30^.

## RESULTS

**Figure 1.**
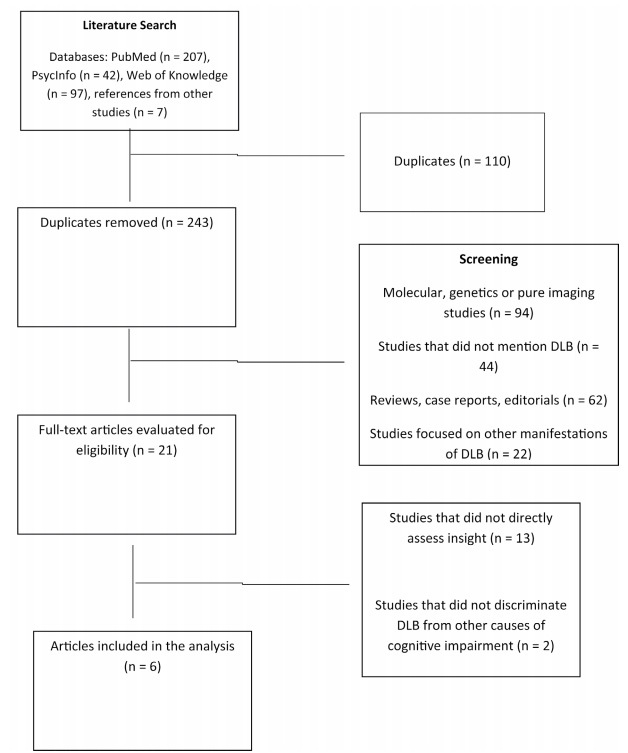
Stages of data search and selection.

Out of 243 studies retrieved through the database and reference searches, six were included in this review. Figure 1 depicts the stages for selection of studies. Among the studies selected, four evaluated anosognosia for objective deficits and two assessed awareness of psychiatric manifestations, namely visual hallucinations and presence phenomena (i.e. the feeling of a presence without any objectively identifiable stimulus). Almost all of the studies had a cross-sectional design, except for one^31^ retrospective cohort study. The articles selected are detailed in Table 1.

**Table 1.** Articles selected.

Study	Object of awareness	Study design	Setting	Number of individuals	DLB diagnostic criteria	Method of anosognosia evaluation	Results	Statistical significance	Quality assessment (Newcastle-Ottawa scale)
Del Ser et al.^31^	Not specified	Retrospective cohort	Pathological evaluation of patients from an outpatient setting	11 pure DLB, 35 pure AD, 18 AD+DLB (pathological signs of both diseases)	Neuropathological examination + DLB consensus criteria (McKeith, 1996)	Not specified; patients were evaluated as either having loss of insight or not	Percentage of patients with loss of insight: DLB: 18% AD: 57% AD+DLB: 66%	p = 0.03	8/9
Hanyu et al.^35^	Memory	Cross-sectional	Outpatient clinic	17 DLB, 63 AD, 56 MCI, 14 VaD	DLB consensus criteria (McKeith 2005)	Difference between the *Everyday Memory Checklist* (EMC) filled by the patient and a caregiver. Patients with anosognosia were defined as having a difference greater than 9	Mean EMC difference DLB: 2.5 ± 5.0 AD: 13.9 ± 9.2 Patients with anosognosia DLB: 6% AD: 65%	p < 0.0001	7/9
Iizuka et al.^32^	Memory	Cross-sectional	Hospital-based study	34 DLB, 18 HC	DLB consensus criteria (McKeith 2005)	Index comparing objective memory loss (RAVLT) and subjective memory loss (MAC-Q)	Mean anosognosia index and standard deviation DLB: -1.514 ± 1.693 HC: 0.000 ± 1.548	p < 0.01	6/9
Rahman-Filipiak et al.^36^	Memory	Cross-sectional	Participants recruited from the community	16 DLB, 186 HC, 24 naMCI, 46 aMCI-SD, 71 aMCI-MD, 129 AD, 16 FTD	DLB consensus criteria (McKeith 2005)	The study did not calculate a specific anosognosia index but compared the scores of the traditional version of MAC-Q (subjective memory loss) and an informant version (MAC-F) across different diagnoses.	MAC-Q: No group differences MAC-F: DLB had worse memory than HC and no significant difference from AD	p < 0.004 for HC p = 0.999 for AD	7/9
Ballard et al.^33^	Visual hallucinations	Cross-sectional	Outpatient clinic	38 DLB, 8 AD*	Operational criteria for senile dementia of Lewy body type (McKeith 1992)	Standardized interview. Insight was classified as absent or partial	Percentage of patients with no insight DLB: 63% AD: 75%	The article did not provide a p-value. OR: 1.7; 95% CI 0.6-66.0	5/9
Reckner et al.^34^	Presence phenomena	Cross-sectional	Outpatient clinic	Cases: 4 DLB, 13 PD with presence phenomena** Controls: 25 PD without presence phenomena	DLB consensus criteria (McKeith, 2017)	Semi-structured interview. Insight was classified as reduced or preserved	Percentage of patients with reduced insight: DLB: 25% PD: 69%	Not described; study did not focus on comparison between etiologies	6/9

*42 patients with DLB and 30 with AD were recruited, but only the number aforementioned had visual hallucinations; **7 patients with DLB and 18 with PD were recruited, but only the number aforementioned filled out the evaluation questionnaire. AD: Alzheimer’s disease; DLB: dementia with Lewy bodies; EMC: Everyday Memory Checklist; HC: healthy controls; MCI: mild cognitive impairment; aMCI-SD: amnestic mild cognitive impairment - single domain; aMCI-MD: amnestic mild cognitive impairment - multiple domains; na-MCI: non-amnestic mild cognitive impairment; PD: Parkinson’s disease; VaD: vascular dementia.

### Sample characteristics

The total sample included in the studies selected comprised 842 patients, among whom 138 had been diagnosed as presenting DLB. Among the studies that evaluated awareness in relation to objective deficits, only one compared DLB with healthy controls^32^, while the others assessed differences between subjects with DLB and other conditions, namely AD, FTD, vascular dementia and mild cognitive impairment (MCI). Among the studies that assessed psychiatric symptoms, patients with DLB were compared with individuals with AD^33^ and Parkinson’s disease (PD)^34^.

Three of the studies provided detailed demographic information^31^^,^^32^^,^^35^. In all of these studies, the mean age of the subjects with DLB was at least 70 years (overall mean: 74.5 years old; SD: 6.4) and the mean education level was at least 12 years (overall mean 12.7 years; SD: 3.1). Ballard et al.^33^ described a mean age of 73.6 years but did not mention either standard deviations or years of schooling. One study^34^ only described overall demographic characteristics but not those concerning the patients who underwent awareness evaluation. Only one study^36^ did not acknowledge the demographic characteristics of the participants. Patients attending outpatient clinics or hospitals were assessed in five of the studies, whereas only one included a community-based sample^36^.

Three studies provided an insight into dementia severity through using the Mini-Mental Status Examination (MMSE): two that evaluated memory anosognosia^32^^,^^35^ (mean: 22.8; SD: 2.7) and one that assessed awareness in relation to hallucinations^33^ (mean: 13.9; SD was not provided).

### Diagnosis

Three clinical criteria were used as the basis for the diagnosis of DLB. In chronological order, this was done for the following studies:

- Ballard et al.^33^: operational criteria for senile dementia of Lewy body type^37^.
- Hanyu et al.^35^, Iizuka et al.^32^, Rahman-Filipiak et al.^36^: Third Report of the DLB consortium^38^.
- Reckner et al.^34^: Fourth Consensus report of the DLB consortium^39^.

One study^31^ used pathological criteria to diagnose DLB. Pure DLB was defined as presence of both of the following: (i) presence of numerous cortical and subcortical Lewy bodies reaching a score of at least 7, according to the consortium rating protocol^40^; and (ii) neuritic senile plaques were below the CERAD stage for AD pathology^41^. In addition, comorbid AD and DLB pathology (AD+DLB) corresponded to cases that: (i) fulfilled the CERAD criteria for AD; (ii) presented at least one neurofibrillary tangle per square millimeter in the neocortex; and (iii) showed cortical Lewy bodies reaching consortium rating protocol scores of 3 to 6 (for limbic type DLB) or more than 7. The criteria of the consensus guidelines of the DLB consortium^40^ were also retrospectively applied, which showed that all patients with a pathological diagnosis of pure DLB met the clinical criteria for either possible DLB (2 patients) or probable DLB (9 patients). Among the individuals with pathological diagnosis of AD+DLB, 5 were further classified as probable DLB, 9 as possible DLB and 4 as non-DLB.

In addition, Hanyu et al.^35^ used myocardial MIBG (123 meta-iodobenzylguanidine) scintigraphy to support the clinical diagnosis.

### Anosognosia evaluation

Both studies assessing awareness of psychiatric symptoms used standardized questionnaires. However, neither of them provided details on the methods of evaluation. In the group that evaluated anosognosia of objective deficits, most of the studies used self-report questionnaires addressing memory complaints. The results were either compared to scores from an informant-based instrument about the patients’ cognitive difficulties or contrasted with the participants’ cognitive performance in neuropsychological tasks^32^. One study, however, used a qualitative approach, assessing level of awareness based solely on the clinician’s impression^31^.

Instruments and further methodological details, as employed in the articles selected, are described as follows: Hanyu et al.^35^ used the Japanese version of the Everyday Memory Checklist (EMC)^42^, which was filled out by both the patient and the caregiver. This questionnaire comprises thirteen problems and the individual is asked to rate the frequency with which they occur. These authors produced an anosognosia index, which consisted of the difference between the patient’s and the caregiver’s scores. They also defined a cutoff value, above which the individual was defined as unaware. Rahman-Filipiak et al.^36^ used the Memory Assessment Complaint Questionnaire (MAC-Q)^43^ and adapted it into an informant version (MAC-F). In MAC-Q, patients compare their current memory regarding everyday tasks to previous years. Options are provided on a five-point Likert-style scale (ranging from “much better now” to “much poorer now”). These authors did not provide an anosognosia index but compared the responses in both questionnaires among different disorders. Iizuka et al.^32^ also used the MAC-Q to assess memory complaints. However, instead of comparing it with the opinions of caregivers, they used an objective memory evaluation, the Rey Auditory Verbal Learning Test. They used the scores to compose an awareness index that was calculated using standardized discrepancies taking into account the mean and standard deviation of normal controls. As mentioned, Del Ser et al.^31^ did not use quantitative methods, but undertook a qualitative clinical evaluation of deficit awareness.

### Anosognosia for cognitive domains in dementia with Lewy bodies

Three studies assessed anosognosia for memory, whereas one^31^ did not specify which cognitive domain was evaluated. The prevalence of anosognosia was described as being higher in AD than in DLB in two studies, one that appraised memory alone (65% versus 6%)^35^ and one that did not specify the object of awareness (57% versus 28%)^31^. Interestingly, in this last study^31^ a group with mixed pathology (AD+DLB) had a higher rate of unawareness than the other two groups (66%; p = 0.03). Conversely, Rahman-Filipiak et al.^36^ reported that no differences between DLB and AD were observed across patient and caregiver responses in memory awareness questionnaires.

In addition, two studies compared DLB patients with healthy individuals. One did not provide an anosognosia index^36^ but showed that, while the two groups had similar scores in the questionnaire on memory complaints that was filled out by the patients, the scores in the questionnaires filled out by the informants were higher in DLB cases. This indicated that awareness in DLB cases was worse than in healthy individuals. Similarly, Iizuka et al.^32^ found that the mean anosognosia index was lower (meaning worse awareness) in patients with DLB than in healthy controls (-1.514 versus 0.000).

Iizuka et al.^32^ also demonstrated that awareness for memory deficits in DLB cases was inversely related to ^18^F-FDG uptake bilaterally in the posterior cingulate cortex and right orbitofrontal cortex. This finding was similar to what had already been demonstrated in individuals with AD^18^. The study also showed that striatal dopamine deficiency, as demonstrated by dopamine transporter (DAT) binding, did not influence memory awareness. On the other hand, there was a direct relationship between the cingulate island sign (CIS) ratio (i.e. sparing of the posterior cingulate cortex relative to precuneus and cuneus) and memory awareness. Given that a lower CIS ratio is associated with larger amounts of neurofibrillary tangles^44^, this finding suggests that co-occurrence of AD-type pathology may be at least partially responsible for anosognosia in patients with DLB.

#### Anosognosia for psychiatric symptoms in dementia with Lewy bodies

Only one study evaluated anosognosia for visual hallucinations in patients with DLB. Ballard et al.^33^ stated that 63% of DLB patients with such symptoms had no awareness of the abnormality of their visions. The difference between anosognosia in these individuals and in AD patients was not statistically significant (OR: 1.7; 95% CI 0.6-66.0). Reckner et al.^34^ found that 25% of the patients with DLB showed anosognosia for presence phenomena. However, they evaluated a very small number of individuals (n = 4) and the study did not compare awareness between DLB and PD directly.

### Quality assessment

Potential risk of biases was detected in all the studies selected. The representativeness of the sample was considered poor in the studies using convenience samples, since they had considered neither the sociodemographic variables (educational level, for example) nor the disease severity, which potentially differs from what is seen among individuals in the community. In addition, two studies provided incomplete data on the sociodemographic characteristics of the participants^33^^,^^34^ and one did not specify these characteristics at all^36^. Comparison bias may have occurred in three studies^33^^,^^34^^,^^36^ that did not control for some of the main confounders (namely age and educational level). Lastly, one of the studies did not specify how anosognosia was assessed and, thus, its findings are not replicable^31^.

## DISCUSSION

Although all the studies indicated that memory anosognosia occurs in DLB, it was not possible to apprehend its prevalence from the evidence available. The main reason for this was the heterogeneity of the methods used for awareness measurement, which prevented comparison between different studies. Moreover, anosognosia has a full spectrum of severity and no precise line separates normal from pathological awareness. For this reason, arbitrary cutoff points were chosen for defining patients as having anosognosia or not.

Nevertheless, it was possible to compare subjects with DLB with other individuals. Patients with DLB had worse awareness for memory difficulties than healthy older controls, but the results concerning differences in the frequency of this phenomenon between DLB and AD patients were inconsistent across studies. Use of different evaluation methods probably also helps to explain the discrepant findings.

More detailed information on the characterization of anosognosia in different dementia subtypes is necessary in order to comprehend the complex relationship between DLB and AD regarding memory awareness. Previous data have demonstrated that pure DLB pathology is not the most common presentation of the disorder, considering that only 23% of patients were found to display “pure” DLB in a neuropathological analysis, in contrast to 30% with concurrent high density of neurofibrillary tangles^45^. Consequently, unravelling the independent contribution of each pathological mechanism for the emergence of anosognosia is a major challenge in this field. Wilson et al.^15^ previously demonstrated that lack of memory awareness was only associated with neurofibrillary tangles (but not amyloid plaques), gross infarcts and TDP-43 pathology, while the presence of Lewy bodies did not significantly correlate with this clinical variable. Conversely, as described in the present review, Iizuka^32^ did not find any correlation between memory-deficit awareness in DLB and striatal dopaminergic activity, whereas cingulate island sign ratio, an indirect biomarker for AD pathology, was associated with anosognosia. This evidence hinted that AD pathology, and not DLB, could account for anosognosia in those cases.

However, one of the studies selected implied that comorbid DLB pathology may in fact augment the decline of self-perception in patients with AD, as subjects with both conditions had higher rates of anosognosia than those with pure AD^31^. A similar finding was described by Peavy et al.^46^ in 2016, in comparing patients with DLB with and without pathological findings of AD. Memory complaints were similar in the two groups, while performance in memory tests was significantly worse in patients with co-occurring AD pathology. Those authors did not formally assess anosognosia, but their findings also suggested that the presence of AD pathological signature worsens memory awareness in patients with DLB.

It should be noted that awareness of memory deficits was analyzed in all the studies selected, except for one article, in which it was not stated which cognitive domains were investigated. Thus, it is not possible to infer whether the relationship between AD pathology and memory anosognosia in patients with DLB also concerns other objects of awareness. Hence, exploration of non-amnestic awareness in DLB in future research could provide interesting insights.

There is little evidence regarding the anatomical basis of memory anosognosia in DLB. Iizuka^32^ found that DLB patients with anosognosia had lower glucose metabolism in the bilateral posterior cingulate cortex and in the right orbitofrontal cortex. This finding is similar to what was previously described among patients with AD and FTD^18^. Nevertheless, since that study implied that AD pathology had a fundamental role in memory anosognosia in patients with DLB, it is not possible to state whether this anatomical substrate is specifically present in DLB or whether it only reflects the presence of AD.

Surprisingly, only one article^33^ formally described awareness of hallucinations in patients with DLB, albeit only among patients with a mean MMSE of 13.9, i.e. probably at an advanced stage of dementia. The control group only included patients with AD, which is a disorder only rarely associated with hallucinations, unlike PD dementia. Besides that, because the study did not focus on awareness, there were no details of measurement methods.

There was not enough evidence to characterize anosognosia for presence phenomena, given that the sole article to evaluate it^34^ focused on PD patients and included only four individuals with DLB.

In addition to the shortcomings listed above, risks relating to selection, comparison or outcome biases were found for all the articles, selected which might limit the generalization of the findings. Moreover, heterogeneity regarding diagnostic methods for DLB across studies (clinical, MIBG and neuropathological assessments), as well as discrepant strategies for approaches to anosognosia (difference between self-reports and collateral reports, discrepancies between self-reports and performance in tasks or clinician’s impression), hampered the comparability of the results across studies. Lastly, the scarcity of studies addressing this clinical entity needs to be highlighted, given that only six studies were found in this review.

## CONCLUSION

Anosognosia is more frequent among patients with DLB than among healthy individuals. However, the evidence is inconsistent in comparison with AD. Co-existence of AD pathology seems to be, at least in part, responsible for anosognosia for memory deficits in DLB. However, because the assessments were limited to memory-impairment awareness, this diminishes the possibility for generalization of this finding to other cognitive domains. It is still unclear whether the presence of multiple pathologies leads to higher prevalence of anosognosia than in cases of pure AD.
